# Supplementary figures and images for: Visualizing sarcomere and cellular dynamics in skeletal muscle to improve cell therapies
Source: eLife. 2024 Dec 17;13:e95597. doi: 10.7554/eLife.95597 (PMC11651650; doi:10.7554/eLife.95597)

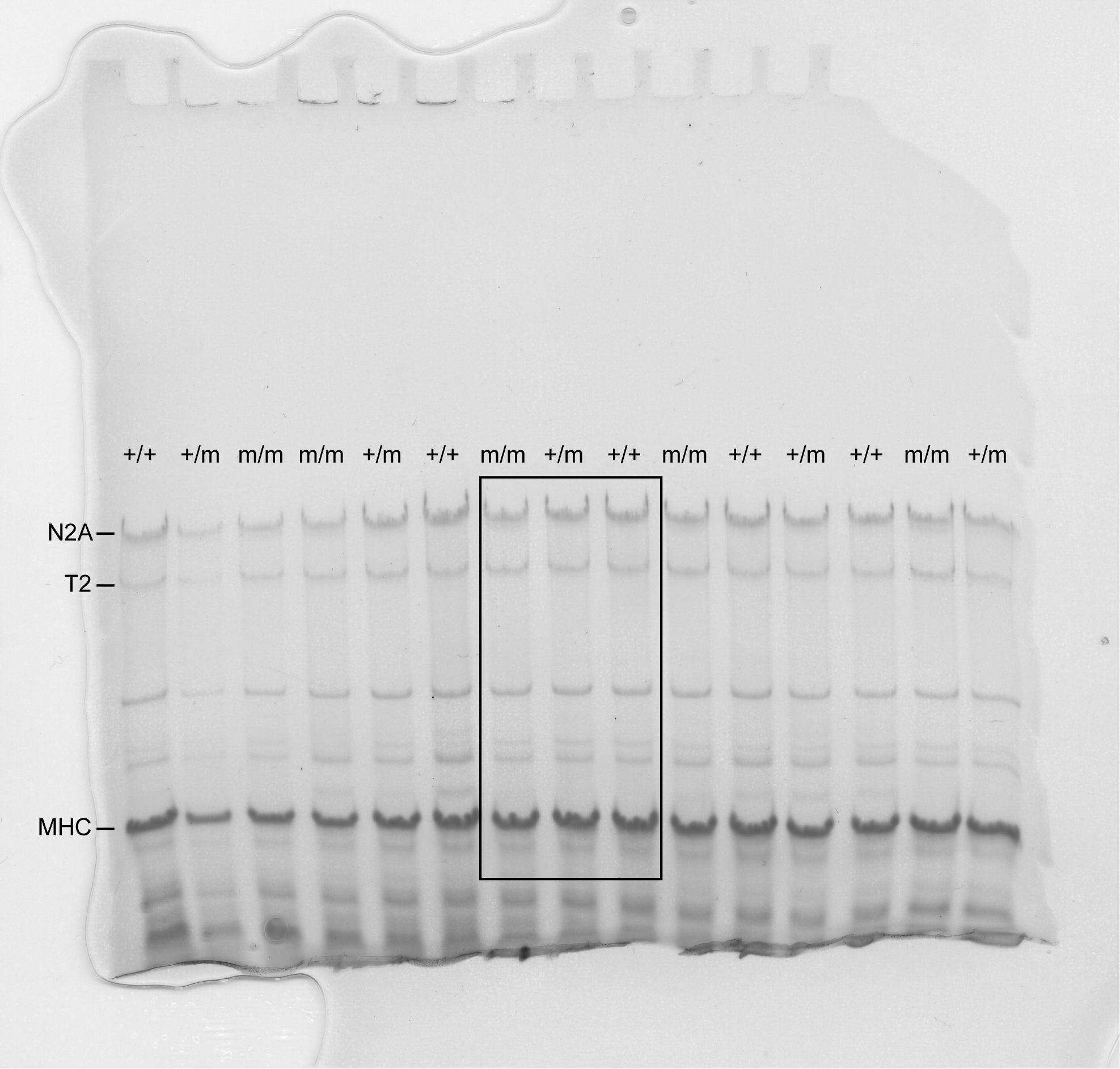

Supplement: Figure 1—figure supplement 1—source data 1. [file elife-95597-fig1-figsupp1-data1.tif]
